# Non Typical Type 1 Diabetes Mellitus Onset in a Child With Salt-Wasting Congenital Adrenal Hyperplasia

**DOI:** 10.1210/jcemcr/luae106

**Published:** 2024-08-01

**Authors:** Federica Rodofile, Francesca Franco, Nicoletta Buccino, Paola Cogo

**Affiliations:** Division of Pediatrics, Department of Medicine, University of Udine, 33100 Udine, Italy; Division of Pediatrics, Department of Medicine, ASUFC Hospital Udine, 33100 Udine, Italy; Division of Pediatrics, Department of Medicine, University of Udine, 33100 Udine, Italy; Division of Pediatrics, Department of Medicine, University of Udine, 33100 Udine, Italy

**Keywords:** type I diabetes, salt-wasting congenital adrenal hyperplasia, CYP21A2, hypoglycemia, cortisol

## Abstract

Type 1 diabetes mellitus (T1DM) and congenital adrenal hyperplasia (CAH) are 2 complex endocrine disorders with neighboring genetic loci. We present a case of T1DM onset in a 6-year-old child, already affected by 21-hydroxylase deficiency (salt-wasting CAH) diagnosed at 18 days of age, who was referred to our clinic because of typical symptoms of diabetes despite nondiagnostic fasting blood glucose values. Further analysis revealed elevated glycated hemoglobin (HbA1c), low C-peptide, and specific autoantibodies suggesting the diagnosis of T1DM. Although he only started with rapid-acting insulin analogue before meals, he presented spontaneous episodes of hypoglycemia just before the morning hydrocortisone dose, due to an underdosed glucocorticoid intake. Based on continuous glycemic monitoring (CGM), his morning dose was increased and given earlier; then we decided to apply an advanced hybrid closed-loop insulin pump to maintain glycemic time in range above 70%. Fasting glucose in CAH patients can be lower due to underdosed glucocorticoid replacement therapy. HbA1c and CGM can help recognize T1DM onset and evaluate the correct dosage of corticosteroid therapy in CAH patients. New studies are needed to understand the therapeutic approach for a more specific treatment in case of coexistence of these diseases.

## Introduction

Type 1 diabetes mellitus (T1DM) and congenital adrenal hyperplasia (CAH) are 2 complex genetic endocrine disorders, both relatively common. CAH, inherited as an autosomal recessive disorder, is characterized by impaired cortisol synthesis and it is classified into classic “severe” (salt-wasting and simple virilizing, diagnosed at birth) and non-classic “mild” (late-onset) types. The incidence of classic CAH in Western populations is about 1:10 000 to 1:15 000 (1).

T1DM is one of the most common chronic diseases of childhood, accounting for nearly 98% of all cases of diabetes in children under 10 years of age and over 87% of all cases between 10 and 19 years; however, the disease can occur at any age (2). Its optimal treatment includes basal and multiple doses of insulin using injections or an insulin pump and the use of specific glycemic monitoring devices to ameliorate the doses of insulin to be administered. Although T1DM and 21-hydroxylase deficiency CAH are diseases that have neighboring genetic loci, both conditions are expected to occur independently of each other (3). We present a case of a child affected by salt-wasting CAH who develops T1DM at 6 years of age.

## Case Presentation

Our patient is a 6-year-old Italian boy who was diagnosed with salt-wasting CAH due to 21-hydroxylase deficiency at 18 days of age after presenting with hypovolemic shock due to adrenal insufficiency. Genetic testing revealed a mutation of the CYP21A2 gene. He inherited the complete paternal CYP21A2 gene deletion and an extended deletion of the third exon from the mother. His CAH was managed with standard doses of hydrocortisone (14 mg/m^2^/day: divided into 3 equal doses given every 8 hours at 8 Am, 4 Pm, 12 Am) and fludrocortisone (0.025 mg once a day) with good family compliance. There was no family history of autoimmune diseases; in particular, there was no one with T1DM. The child was referred to our clinic because of polyuria, polydipsia, polyphagia, and weight loss in a 2-week period prior to hospitalization. His pediatrician had already prescribed blood tests, which included normal fasting glucose value (87 mg/dL; 4.87 mmol/L; normal reference range 60-100 mg/dL; 3.9-5.6 mmol/L) and stick urine tests which showed the presence of glycosuria and ketonuria. At the moment of hospitalization, the child had a weight of 21.6 kg (40 °centile for age and sex) and a height of 114 cm (12° centile for age and sex). Body mass index was 16.7 kg/m^2^ (78° centile for age and sex).

## Diagnostic Assessment

At the time of admission, 5 days after the previous analysis, he presented with a normal blood pressure and mild signs of clinical dehydration. Initial laboratory evaluation revealed a fasting blood glucose not diagnostic for diabetes (122 mg/dL; 6.77 mmol/L; normal reference range 60-100 mg/dL; 3.9-5.6 mmol/L) although with persistent significant amounts of glycosuria (>1000 mg/dL, 55.56 mmol/L; normal reference 0-15 mg/dL; 0-0.8 mmol/L) and ketonuria (20 mg/dL; 3.44 mmol/L, normal reference range value <5 mg/dL; < 0.86 mmol/L); suspecting T1DM, we performed first-level testing (Table 1). Diagnosis of T1DM was subsequently confirmed by high levels of glycated hemoglobin (HbA1c) and presence of associated autoantibodies (Table 1). Blood studies related to CAH showed a high renin level (>500 μUI/mL, >7.1 pmol/L; normal reference range, 3.5-46.0 μUI/mL, 0.05-0.65 pmol/L), normal electrolytes, a slightly increased ACTH (60 pg/mL; 13.21 pmol/L; normal reference range, 5-49 pg/mL; 1.1-10.8 pmol/L) and elevated 17-hydroxyprogesterone (17-OHP) (9.4 ng/mL; 28.4 nmol/L; normal reference range, <1.1 ng/mL; <3.32 nmol/L), with other androgens in the normal range.

**Table 1.** Urine and blood tests at the admission including hormone and diabetes autoimmunity tests

	Performed tests	Conventional value (SI Value)	Normal reference range (SI normal range)
Blood tests	Fasting blood glucose	122 mg/dL (6.77 mmol/L)	60-100 mg/dL (3.9-5.6 mmol/L)
	Glycated hemoglobin (HbA1c)	11.6%	<6.5%
	Insulin C-peptide	<0.81 ng/mL (0.27 nmol/L)	0.90-7.00 ng/mL (0.3-2.3 nmol/L)
	Serum insulin level	<2 μUI/mL (<13.8 pmol/L)	3.00-25.00 μUI/mL (20.8-173.6 pmol/L)
	pH (blood)	7.42	7.35-7.45
	HCO_3_^−^	25.1 mEq/L (25.1 mmol/L)	22-26 mEq/L (22-26 mmol/L)
	Base excess	0.8 mEq/L (0.8 mmol/L)	0 ± 2 mEq/L (0 ± 2 mmol/L)
Urine tests	pH (urine)	6	6-8
	Urine ketones	20 mg/dL (3.44 mmol/L)	<5 mg/dL (0.86 mmol/L)
	Urine glucose	>1000 mg/dL (55.56 mmol/L)	0-15 mg/dL (0-0.8 mmol/L)
Hormones	Renin	>500 μUI/mL (>7.1 pmol/L)	3.5-46.0 μUI/mL (0.05-0.65 pmol/L)
	ACTH	60 pg/mL (13.21 pmol/L)	18 019 pg/mL (1.1-10.8 pmol/L)
	Cortisol	27 μg/dL (745 nmol/L)	5.4-23.5 μg/dL (150-650 at 8.00 Am nmol/L)
	17-hydroxyprogesterone	9.4 ng/mL (28.4 nmol/L)	<1.1 ng/mL (<3.32 nmol/L)
	Testosterone	<20 ng/dL (<0.69 nmol/L)	200-814 ng/dL (6.9-28.1 nmol/L)
	Dehydroepiandrosterone sulfated	<15 μg/dL (0.4 μmol/L)	15-100 μg/dL (40-271 μmol/L)
	Androstenedione	0.30 ng/mL (1.05 nmol/L)	0.30-3.30 ng/mL (1.05-11.5 nmol/L)
Autoimmunity	Insulinoma-associated-2 autoantibodies	(4000.00 UI/mL)	(0-10 UI/mL)
	Zinc transporter-8-autoantibody	(133 U/mL)	(0-15 U/mL)
	Antibodies to glutamic acid decarboxylase	(<5.00 UI/mL)	(0-10 UI/mL)
	Tissue transglutaminase IgA antibody	2 (AU/mL)	<10 AU/mL

## Treatment

As a first line of treatment, he was hydrated with normal saline and then started therapy with subcutaneous insulin. His history was negative for recent infections or fever and his corticosteroid therapy had not been changed recently. Because of dehydration and considering the onset of diabetes as a “stressful” condition, we decided to double hydrocortisone therapy for 2 days. At the admission, celiac disease antibodies were examined, with a negative result (Table 1). Furthermore, genetic tests were performed for susceptibility to diabetes, which showed HLA DRB1*04, *07, DQA1*02, *03, and DQB1*02:02, *03;02 and definition of risk of celiac disease with DQ2 (DQB1*02) and DQ8 (DQA1*03, DQB1*03;02).

**Figure 1.**
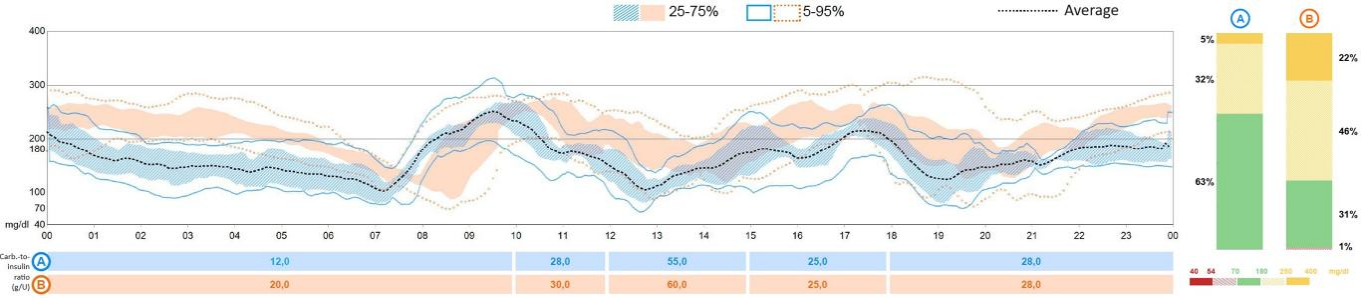
A represents the use of the same pump in automatic mode (AHCL). B represents the glucose trend in the first week with an insulin pump in manual mode. Columns represent glycemic ranges: green is time in range (TIR) equals to 70–180 mg/dL. Time above range (TAR), > 250 mg/dL, is represented in yellow while time below range (TBR) is represented in red (< 54 mg/dL).

At the beginning, it was decided to use only a rapid-acting insulin analogue before meals and for corrections of hyperglycemia without introducing basal insulin. However, during hospitalization, he presented episodes of hypoglycemia (down to 30 mg/dL; 1.66 mmol/L) and spontaneous glycemic decreases, particularly just before the morning hydrocortisone administration (Fig. 1). Suspecting insufficient cortisol replacement as an insulin counterregulatory hormone, and the difficulty to maintain stable glycemic values, it was decided to apply insulin pump (Minimed 780G) in manual mode for the first week and then in automatic mode, an advanced hybrid closed-loop system (AHCL). AHCL systems differ from the standard by providing autocorrection insulin boluses to help correct high sensor glucose readings with better glycemic outcomes (Fig. 1).

## Outcome and Follow-Up

**Figure 2.**
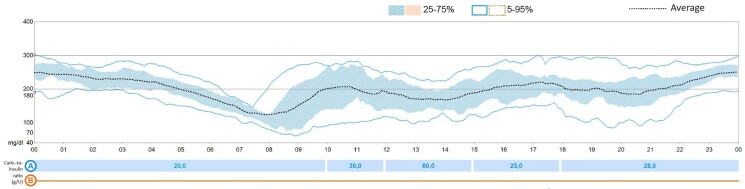
Glucose trend at the beginning with rapid-acting insulin analogue before meals without basal insulin. Spontaneous glycemic decrease before the morning hydrocortisone administration.

Based on continuous glycemic monitoring (CGM), the morning dose of hydrocortisone was increased and given earlier (16 mg/m^2^/day: 4 mg at 12 Am + 6 mg at 6.30 Am + 4 mg at 4 Pm). These variations and the use of advanced insulin devices allowed a glycemic time in range above 70%, as suggested by guidelines (4) (Fig. 2).

## Discussion

We report a case of T1DM in a 6-year-old child with a previous diagnosis of salt-wasting CAH. The presence of both T1DM and salt-wasting CAH is a rare event scarcely reported in the literature (Table 2).

**Table 2.** Review of literature: case reports of coexistence of congenital adrenal hyperplasia and type-1-diabetes mellitus

Case report	CAH diagnosis (year)	T1DM diagnosis (year)	Comorbidity
Islam et al. (3)	at birth	11 yo at routine exams (plasma glucose of 596 mg/dL)	Brother with CAH, no family history for autoimmune diseases
Islam et al. (3)	2 weeks after birth.	2 weeks after birth at routine exams (blood glucose level 710 mg/dL)	No familial history both for CAH and T1DM
	Positive neonatal screening		
Aureli et al. (10)	6.5 y/o (non classical CAH)	16 y/o, diabetic ketoacidosis (plasma glucose of 382 mg/dL)	Prader–Willi syndrome, central precocious puberty, no family history for autoimmune diseases
Zachariah et al. (11)	4 y/o (non classical CAH)	15 y/o, diabetic ketoacidosis (plasma glucose of 55 mg/dL)	-

T1DM is a multifactorial disease with the major susceptibility locus mapping to the HLA class II genes at chromosome 6p21.3, specifically HLA- DQA1, DQB1, and DRB1 genes, located on chromosome 6p21.32. The location of the CYP21A2 gene is in close proximity to the HLA B and HLA DR loci but both conditions are expected to occur independently of each other.

CAH is caused mostly by a decrease or absence of 21-hydroxylase enzyme, and it is a Mendelian condition known to be inherited in an autosomal recessive manner. CAH due to 21-hydroxylase deficiency is associated with biallelic mutations in the CYP21A2 gene, at chromosome 6p21.33, a location which is in close proximity to the HLA B and HLA DR loci (3); Jayakrishnan et al showed an association between CYP21A2 (point mutation p.I172N), and the HLA-DQB1*03 allele which was mutated in our case (5). Our patient received CYP21A2 gene deletion from both of the parents and he also had genetic susceptibilities to T1DM and celiac disease. At the time of admission, the child also underwent screening for celiac disease, resulting negative. Falhammar et al suggest that patients with 21-hydroxylase deficiency had a higher risk of developing autoimmune disorders; in particular, in the Swedish adult population, women with a specific CAH phenotype have an increased risk for T1DM. The glucocorticoid replacement could be assumed to have an immunomodulating effect but a supraphysiological dose increases the risk of the diseases associated with metabolic-related manifestations (ie, cardiovascular disease and type 2 diabetes mellitus) (6). Several studies have shown that hypoglycemia is present in children with CAH not only during severe acute illnesses or infectious episodes but also in mild diseases, despite high doses of hydrocortisone replacement (7).

The inability to produce cortisol, as an insulin counterregulatory hormone, and an insufficient glucocorticoid replacement dose could explain low blood glucose values at the onset of diabetes (87 mg/dL; 4.87 mmol/L; normal reference range 60-100 mg/dL; 3.9-5.6 mmol/L). In a stressful situation, like the onset of T1DM, a subject with an intact, functional, adrenal gland would secrete higher doses of cortisol, laying the foundations for a higher glycemic response. This reasoning would explain why it was necessary to increase the dosage of hydrocortisone.

During his hospitalization, the patient presented episodes of hypoglycemia and spontaneous decreases in glucose levels, particularly just before the morning hydrocortisone dose even in the absence of basal insulin administration. Therefore, considering the absence of basal insulin, it was not possible to modulate insulin delivery but was only possible to act on hydrocortisone modulation. After pump placement, blood glucose was better controlled with a higher dose of hydrocortisone.

In a case report by Xiang et al, steroid dose and glucose levels were investigated using CGM in a 29-year-old patient with CAH. They observed that blood glucose levels during the night and fasting periods decreased considerably before glucocorticoid administration, while they increased significantly postprandially after cortisol treatment (8). Cortisol deficiency increases insulin sensitivity, activates gluconeogenesis, decreases glycogenolysis, and enhances the activity of counterregulatory hormones such as glucagon, epinephrine and other catecholamines (9). The inability to produce cortisol, as an insulin counterregulatory hormone, causes less glycogenolysis, which explains the requirement of less insulin and the possibility of manifesting fasting blood glucose in range at the onset of what will turn out to be a diagnosis of T1DM. Therefore, in our patient, initial laboratory evaluation revealed elevated levels of renin associated with mild signs of dehydration. In untreated or undertreated salt-wasting CAH, hyperreninemia is a common finding due to mineralocorticoid deficiency, whereas it is not common in adequately treated patients. Despite stable replacement therapy, our patient presented elevated plasma renin levels. This finding is best explained by his mild dehydration, due to the onset of T1DM, and activation of the renin-angiotensin-aldosterone (RAAS) system.

CAH and T1DM are 2 conditions with a substantial impact on the patient. In our case, normal fasting blood glucose levels masked the onset of diabetes. It is important, in patients affected by both of these conditions, to conduct further examinations to avoid a delay in diagnosis. The use of CGM helped to increase and/or move to an earlier time the usual morning dose of hydrocortisone while the use of an advanced hybrid closed-loop (AHCL) insulin pump was foundational to reduce glycemic variability and improve glycemic control.

## Learning Points

- T1DM and CAH are 2 complex endocrine disorders with neighboring genetic loci. These conditions are expected to occur independently of each other, but in case of concomitance, therapy of each influences the other.
- Fasting glucose levels in CAH patients may not be diagnostic of diabetes; however, HbA1c and glycemic trends could be useful to recognize the onset of T1DM.
- Continuous glucose monitoring allows for the determination of the correct dosage and timing of corticosteroid therapy in CAH. In these patients, the use of continuous glycemic monitoring and an advanced hybrid closed-loop (AHCL) insulin pump is fundamental to reduce glycemic variability and improve glycemic control.
- New studies are needed to understand the best therapeutic for a better diagnosis and more specific treatment approaches in CAH patients with a diagnosis of T1DM.

## Contributors

All authors made individual contributions to authorship. F.R. and F.F. were involved in the diagnosis and management of this patient and manuscript submission. N.B. was involved in literature research. F.F. and P.C. held the paramount clinical decision-making role during the patient’s hospitalization. All authors reviewed and approved the final draft.

## Data Availability

Original data generated and analyzed for this case report are included in this published article.

## Abbreviations

AHCL: advanced hybrid closed loop
CAH: congenital adrenal hyperplasia
CGM: continuous glucose monitoring
HbA1c: glycated hemoglobin
T1DM: type 1 diabetes mellitus
